# A Comparison of Single- and Multi-Echo Processing of Functional MRI Data During Overt Autobiographical Recall

**DOI:** 10.3389/fnins.2022.854387

**Published:** 2022-04-25

**Authors:** Adrian W. Gilmore, Anna M. Agron, Estefanía I. González-Araya, Stephen J. Gotts, Alex Martin

**Affiliations:** Laboratory of Brain and Cognition, National Institute of Mental Health, National Institutes of Health, Bethesda, MD, United States

**Keywords:** autobiographical memory, fMRI, hippocampus, multi-echo fMRI, time

## Abstract

Recent years have seen an increase in the use of multi-echo fMRI designs by cognitive neuroscientists. Acquiring multiple echoes allows one to increase contrast-to-noise; reduce signal dropout and thermal noise; and identify nuisance signal components in BOLD data. At the same time, multi-echo acquisitions increase data processing complexity and may incur a cost to the temporal and spatial resolution of the acquired data. Here, we re-examine a multi-echo dataset previously analyzed using multi-echo independent components analysis (ME-ICA) and focused on hippocampal activity during the overtly spoken recall of recent and remote autobiographical memories. The goal of the present series of analyses was to determine if ME-ICA’s theoretical denoising benefits might lead to a practical difference in the overall conclusions reached. Compared to single-echo (SE) data, ME-ICA led to qualitatively different findings regarding hippocampal contributions to autobiographical recall: whereas the SE analysis largely failed to reveal hippocampal activity relative to an active baseline, ME-ICA results supported predictions of the Standard Model of Consolidation and a time limited hippocampal involvement. These data provide a practical example of the benefits multi-echo denoising in a naturalistic memory paradigm and demonstrate how they can be used to address long-standing theoretical questions.

## Introduction

Naturalistic fMRI paradigms seek to improve our understanding of the neural bases of “everyday” behavior and strive to be less artificial than more traditional laboratory paradigms (e.g., Hasson and Honey, 2012; Haxby et al., 2020). Naturalistic experiments might involve scanning participants while they watch or describe a popular television show (Chen et al., 2017), read complex narrative passages in the scanner (Finn et al., 2018), or engage in spontaneous conversation with another individual (Jasmin et al., 2019). Although naturalistic paradigms offer opportunities to study brain-behavior relationships beyond those observable in more tightly controlled paradigms, they can also pose additional challenges.

For studies involving spoken responses, the basic act of speaking in an fMRI environment represents a potential issue. Speech will necessarily produce head motion, and if speech is continuous (e.g., Chen et al., 2017; Jasmin et al., 2019) then approaches that require responses between volume acquisitions (e.g., Gracco et al., 2005) or censor specific frames containing speech data (Siegel et al., 2013) are not applicable. Instead, one might be better served by applying recent fMRI timeseries denoising approaches, such as multi-echo independent components analysis (ME-ICA), to remove nuisance signal from one’s data [see Caballero-Gaudes and Reynolds (2017)].

Multi-echo-ICA involves decomposing the multi-echo timeseries into ICA components that are identified as “BOLD-like” and “noise-like,” combining the signal across multiple echoes in each TR, and subsequently regressing the noise-like timeseries identified in ICA from the combined data [see Kundu et al. (2012, 2013, 2017), Gonzalez-Castillo et al. (2016), and Power et al. (2018)]. Although ME-ICA is employed to improve overall data quality, its practical benefit should be balanced against its costs. It requires an up-front decision to collect multiple echoes and the expertise to design and implement specific sequences that allow for it. It will also increase the complexity of one’s preprocessing pipeline and involves steps that may not be implemented in all analysis packages. The additional time required to collect multiple echoes effectively results in slower TRs [typically on the order of 10%, but the cost will depend on the specific number of echoes and echo times (TEs) selected]. Offsetting this TR increase can be accomplished with an increased voxel size, reduction in overall coverage, or through added acceleration and/or multiband.

In this report, the practical benefits of ME-ICA processing are assessed using a recently acquired naturalistic dataset intended to study human memory function (Gilmore et al., 2021a). Forty participants freely and overtly recalled recent and remote autobiographical events for periods of approximately 2 min while undergoing fMRI. As a control task, participants were asked to verbally describe complex photographs. One notable finding from these data was evidence supporting a temporally graded and time-limited role of the hippocampus in the recall of autobiographical memories—an issue that has been discussed at length with several established “camps” in the literature [for recent reviews of various hypotheses, see Squire et al. (2015), Barry and Maguire (2019), Yonelinas et al. (2019), and Gilboa and Moscovitch (2021)]. Overt recall was employed to provide experimental knowledge of the type of information being retrieved during recall (Gilmore et al., 2021b) as this, along with the age of a recalled memory (which itself can also be more easily ascertained using overt recall), appears to be a critical variable in the debate of hippocampal contributions to remote retrieval. The present report revisits these data to ask a practical question: was ME-ICA denoising necessary to observe the differences found during this task? Or, stated differently, would we have obtained the same results without using a multi-echo approach? In the present report, results of the ME-ICA processed data were therefore compared to a standard single-echo (SE) processing stream (Table 1). This comparison was possible because the middle echo of the fMRI data was matched for typical scan acquisition parameters used in any number of memory studies (TE, voxel size, etc.) without the need for additional acceleration that would have impaired the SE data quality.

**TABLE 1:** Summary of analysis pipelines compared in this report.

Standard Processing (2nd echo only)	ME-ICA denoising (three echoes)
First four frames removed	First four frames removed for each TE
Timeseries despiked	Timeseries despiked for each TE
Slice-time corrected	Slice-time corrected for each TE
Volume registration (rigid body)	Volume registration for each TE
	ICA denoising: identifies “BOLD-like” and “noise-like” components across TEs
	“Optimally combined” linear combination of TEs weighted by each voxel’s T_2_*
	“Noise-like” components regressed from the optimally combined data
EPI registered to MP-RAGE	Optimally combined data registered to MP-RAGE
Timeseries data smoothed and converted to % signal change	Timeseries data smoothed and converted to % signal change
Data registered to Talairach atlas (TT_N27)	Data registered to Talairach atlas (TT_N27)
GLM-based mass univariate analysis	GLM-based mass univariate analysis

## Materials and Methods

### Participants

Data for this experiment were taken from a previously published lab dataset (Gilmore et al., 2021a,b). Participants consisted of 40 right-handed young adult participants (23 female; mean age = 24.2 years old) who were native English speakers with normal or corrected-to-normal vision and reported no history of psychiatric or neurologic illness. Informed consent was obtained from all participants and the experiment was approved by the NIH Institutional Review Board (clinical trials number NCT00001360). Participants received monetary compensation for their participation.

### Stimuli

Stimuli consisted of 48 photographic images depicting people engaged in various activities. Images were sized at 525 × 395 pixels (screen resolution: 1,920 × 1,080 pixels) and presented against a black background. Stimuli were presented using PsychoPy2 software (Peirce, 2007; RRID: SCR_006571) on an HP desk- top computer running Windows 10.

### Autobiographical Recall Task

In this task, participants retrieved and described autobiographical memories in response to photographic picture cues. For each trial, participants were first directed to recall an event from one of three different time periods [earlier in the same day (“Today”), 6–18 months prior, or 5–10 years prior]. Participants were given a choice of two photographic cues and had 11 s to select the picture they preferred, which they indicated via button press response. The screen was replaced with a fixation cross once a response was made, and at the end of the selection period an enlarged version of the selected image was presented in the center of the screen for 5 s. Participants used this period to think back to a specific autobiographical event.

Following picture presentation, participants were given 116 s to describe an event while a white fixation cross was presented centrally. Participants were instructed to describe each event in as much detail as possible for the full duration of each trial. Additional task details are described in Gilmore et al. (2021a). A 2.2 s red fixation cross indicated the end of each trial, and trials were separated by a 19.8 s fixation period. One trial from each of the three time periods was included in each Autobiographical Recall task scan run.

Participants were given practice with the task before scanning, and if the events described were not specific, participants were re-instructed and given further practice until specific episodes were being described. During this time, participants were also instructed not to repeat event descriptions in the experiment.

### Picture Description Control Task

As an active control task, participants described events being depicted in cue photographs. The trial timing and structure was identical to that used for the autobiographical recall task, except participants were instructed during the 5 s picture display period to scrutinize the image so that they could describe it in as much detail as possible when it was removed, rather than use it to recall a memory. As before, trials were separated by 19.8 s of fixation, and three trials were included per picture description control task run.

### Audio Recording, Transcript Scoring, and Alignment of Spoken Responses to the BOLD Timeseries

The processing steps associated with the recording and scoring of spoken responses have been described in detail previously (Gilmore et al., 2021a,b). Briefly, recorded audio was transcribed and scored for content using an adapted form of the Autobiographical Interview (Levine et al., 2002; Gaesser et al., 2011). This procedure separates “Internal” (episodic) details specific to the event details from other types of “External” details. Subcategories of Internal details included: Activities, Objects, Perceptual, Person, Place, Thought/Emotion, Time, and Miscellaneous. External detail types included Episodic (i.e., details from other events), Repetitions, Semantic statements, and a catch-all “Other External” category. Timestamps for each spoken word and phrase were generated and matched with the text in transcripts, and different categories of recalled content were converted into event-related regressors for fMRI data analysis, as will be described below.

### fMRI Data Acquisition

Data were acquired on a General Electric Discovery MR750 3.0T scanner, using a 32-channel phased-array head coil. Functional images were acquired using a BOLD-contrast sensitive multi-echo echo-planar sequence [Array Spatial Sensitivity Encoding Technique (ASSET) acceleration factor = 2, TEs = 12.5, 27.6, and 42.7 ms, TR = 2,200 ms, flip angle = 75^°^, 64 × 64 matrix, in-plane resolution = 3.2 mm × 3.2 mm]. Whole-brain EPI volumes (MR frames) of 33 interleaved, 3.5 mm-thick oblique slices were obtained every 2.2 s. Slices were manually aligned to the AC-PC axis. A high-resolution T1 structural image was also obtained for each subject (TE = 3.47 ms, TR = 2.53 s, TI = 900 ms, flip angle = 7^°^, 172 slices of 1 mm × 1 mm × 1 mm voxels).

Foam pillows were provided for all participants to help stabilize head position and scanner noise was attenuated using foam ear plugs and a noise-canceling headset. This headset was also used to communicate with the participant during their time in the scanner. Heart rate was recorded via a sensor placed on the left middle finger and a belt monitored respiration.

### fMRI Processing: Single-Echo (Standard) Analysis

fMRI data were processed following a standard SE preprocessing routine in AFNI (RRID: SCR_005927) to reduce noise and facilitate across-subject comparisons. This processing stream used the 2nd collected echo (TE = 27.6 ms), as this TE is within the typical range SE fMRI paradigms use to study autobiographical recall (e.g., Abraham et al., 2008; Weiler et al., 2010; Gilmore et al., 2018; St Jacques et al., 2018), including those focusing on the MTL or hippocampus (e.g., Svoboda and Levine, 2009; Bonnici and Maguire, 2012; Thakral et al., 2020). Steps included removal of the first four frames to remove potential T1 equilibration effects (*3dTcat*), despiking to remove large transients in the timeseries (*3dDespike*), slice-time correction (*3dTshift*) and frame-by-frame rigid-body realignment to the first volume of each run (*3dvolreg*). Data from each scan run were blurred with a 4 mm FWHM smoothing kernel to approximate the smoothness and minimum cluster extents required to maintain a corrected *p* < 0.05 for whole-brain effects in the ME-ICA pipeline (*k* ≥ 17 for SE data; *k* ≥ 18 for ME-ICA data, see *Voxelwise analysis of temporal distance effects)*. Data were registered to each individual’s T1 image, normalized by the grand mean of each run, and then resampled into 3-mm isotropic voxels and linearly transformed into Talairach atlas space.

### fMRI Processing: Multi-Echo ICA

Multi-echo data were also preprocessed using AFNI, using the same procedures described in previous publications of these data (Gilmore et al., 2021a,b). Initial steps for each TE of each run were identical to those used in the SE processing stream included a removal of the first four frames, despiking, slice-time correction, and rigid-body volume registration. Following these initial steps, data from the three echoes acquired for each run were used to remove additional noise using ME-ICA (Kundu et al., 2012) as implemented in the *meica.py* AFNI function.

This procedure initially calculates a weighted average of the different echo times (“optimally combined” data), which serves to increase contrast-to-noise, reduce signal dropout due to susceptibility artifact, and reduce thermal noise within each voxel. The resulting image is also registered to the corresponding anatomical scan. Separately, the individual echo timeseries data are submitted to an ICA, and the known properties of T_2_* signal decay over time (across the echoes) are used to separate putatively neural components from artifactual components, such as thermal noise or head motion (Power et al., 2018). To be retained, components must show a strong fit with a model that assumes a temporal dependence on signal intensity and also a poor fit with a model that assumes temporal independence (Kundu et al., 2012). Components determined to be noise are then regressed from the optimally combined data. Selection criteria were left at the default settings of AFNI’s *tedana.py* function. Across all included runs, an average of 74.7 (±10.9) components were identified following decomposition and 41.3 (±10.8) were excluded, resulting in an average 168.7 (±10.8) nominal degrees of freedom per run. Following ME-ICA processing, data were spatially blurred with a Gaussian kernel 3 mm full-width at half-maximum, normalized by the grand mean of each run, and then resampled into 3-mm isotropic voxels and linearly transformed into Talairach atlas space, as in the SE pipeline.

### Temporal Signal-to-Noise Comparison

One means of assessing fMRI data quality is to compute its temporal signal-to-noise (tSNR). tSNR is calculated by dividing a timeseries mean by its standard deviation, and will be impacted by nuisance signals related to motion, physiological noise, etc. (Triantafyllou et al., 2011). Thus, one means of appreciating the impact of various processing steps is to compare tSNR. Timeseries tSNR from the SE pipeline were compared to the same data after the optimal combination step in the ME-ICA pipeline (i.e., before any ICA denoising was conducted) as well as on the ICA-denoised ME-ICA data. In all cases, tSNR was calculated prior to smoothing, rescaling, and detrending.

### General Linear Model Creation

Data from both the SE and ME-ICA streams were linearly detrended and analyzed in AFNI using the same general linear model (GLM) approach (*3dDeconvolve*). The initial picture selection period was modeled using a single HRF across all trial types convolved with a boxcar of 11 s duration. The subsequent Picture Display period was also modeled with a single HRF convolved with a boxcar of 5 s duration. The analysis of recall effects utilized a mixed block/event related design (Visscher et al., 2003). Separate regressors modeled sustained effects related to the narration periods of the Autobiographical Recall and Picture Description narration periods. These convolved an HRF with a boxcar of 118.2 s duration in all cases. Additional regressors coded for transient effects associated with each of the 12 types of detail derived from the Autobiographical Interview scoring procedure as described above. Six motion parameters (three translational, three rotational) were included in each subject’s GLM as regressors of non-interest.

### Comparing Single-Echo and Multi-Echo-ICA Effects Within the Hippocampus

Anterior and posterior regions of the hippocampus were defined for each participant, using the uncal apex as a landmark as described previously (Gilmore et al., 2021a). Activity within each hippocampal ROI was averaged across voxels for each condition, using the Picture Description control task as a baseline.

To determine the effect of the processing pipeline on the observed activity differences related to the temporal distance of each event, a repeated measures ANOVA was constructed. This included factors of temporal distance (two levels: Today, 5–10 years ago), subregion (two levels: anterior, posterior), hemisphere (two levels: left, right), and processing pipeline (two levels: SE, ME-ICA). Pairwise comparisons were conducted to characterize significant interactions when appropriate.

Activity in each region, for each pipeline, and each temporal distance was compared against the Picture Description baseline task, using one-sample *t*-tests. Due to the large number of comparisons involved, correction for multiple comparisons included a Bonferroni approach as well False Discovery Rate (FDR). The latter was performed in matlab using *fdr_bh.m*.^1^

### Voxelwise Analysis of Temporal Distance Effects

To test for effects of the recency or remoteness of a memory on retrieval-related BOLD activity, a voxelwise whole brain contrast (paired-samples, two-tailed) of the Today and 5–10 year ago conditions was performed on the SE and ME-ICA pipeline data for each subject. Correction to a whole brain *p* < 0.05 was achieved by requiring a voxelwise *p* < 0.001 and a minimum cluster extent of 17 voxels for the SE data and 18 for the ME-ICA data, determined for each pipeline using AFNI’s *3dClustSim* and its non-Gaussian (-*acf*) autocorrelation function (Cox et al., 2017). Both pipelines identified large (< 800 voxel) posterior midline clusters containing three distinct local maxima. Center of mass coordinates were identified for each location by incrementing the voxelwise *t*-statistic threshold in steps of 0.1 until the three clusters were separated. This was achieved at *t* > 5.04 for the SE data and *t* > 5.36 for the ME-ICA data.

## Results

**FIGURE 1:**
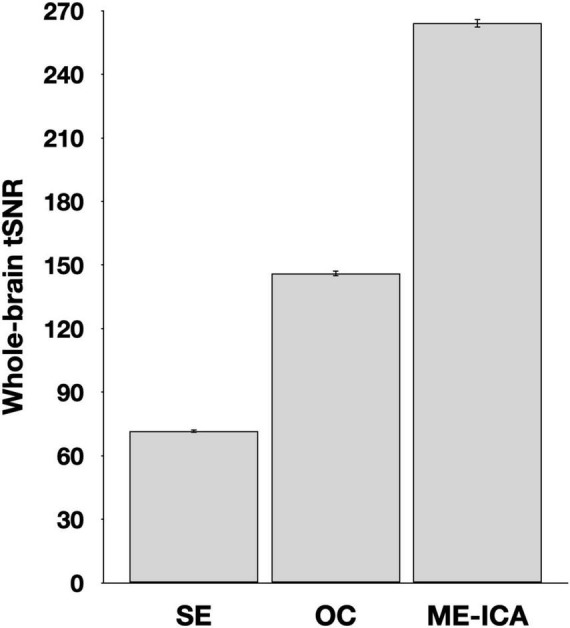
Whole brain temporal signal-to-noise (tSNR) estimates following various stages of processing. Values reflect the timeseries mean divided by its standard deviation prior to smoothing, rescaling, or detrending. Error bars reflect standard error of the mean. SE, single-echo; OC, optimally combined; ME-ICA, multi-echo independent components analysis.

The effectiveness of denoising was estimated by comparisons of tSNR for SE data, the multi-echo data following the optimal combination step (but before ICA denoising was applied), and after ICA denoising was applied in the ME-ICA data (Figure 1). A repeated measures ANOVA identified a significant effect of processing type, *F*_(2,78)_ = 3735.8, *p* < 0.001. Pairwise comparisons identified a significant increase from the SE to the optimally combined data, *t*_(39)_ = 48.3, *p* < 001, as well as from the optimally combined to the fully ME-ICA denoised, *t*_(39)_ = 71.3, *p* < 0.001.

**FIGURE 2:**
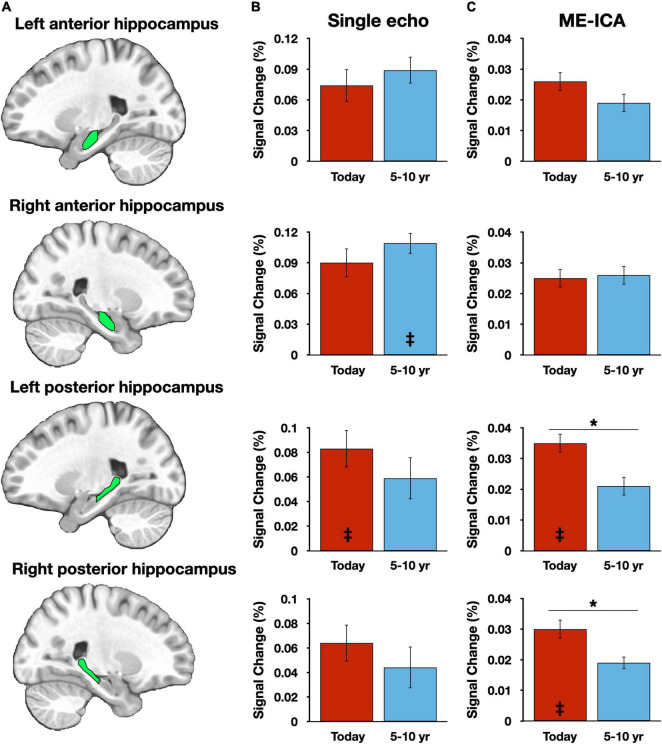
Hippocampal results vary across preprocessing streams. **(A)** Graphic depictions of each manually defined hippocampal subregion. **(B)** Magnitude estimates from the single-echo (SE) analysis pipeline, plotted against the Picture Description control task. **(C)** Magnitude estimates from the multi-echo independent components analysis (ME-ICA) pipeline, plotted against the Picture Description control task. Asterisks (*) denote *p* < 0.05. Inset double daggers signify a significant difference from the Picture Description control task (corrected for multiple comparisons). Error bars reflect within-subject standard error.

Within the hippocampus, anterior and posterior hippocampal subregions (Figure 2A) were manually defined for each participant as regions of interest (ROIs) and activity in each condition was averaged for all voxels within each ROI (Figures 2B,C). A repeated measures ANOVA, with factors of temporal distance (two levels: Today, 5–10 years ago), subregion (two levels: anterior, posterior), hemisphere (two levels: left, right), and pipeline (two levels: SE, ME-ICA) was employed. There was a significant main effect of pipeline, *F*_(1,39)_ = 6.83, *p* = 0.013, reflecting the larger BOLD response magnitudes for the SE processed data. This is consistent with prior investigations comparing SE and ME-ICA processing, which have observed attenuated BOLD signal change estimates despite the overall improvement in contrast-to-noise ratios (Gonzalez-Castillo et al., 2016). No other significant main effects were observed (*p*s ≥ 0.276). Consistent with a prior report using these data (Gilmore et al., 2021a), there was a single two-way interaction of subregion and temporal distance, *F*_(1,39)_ = 10.87, *p* = 0.002, reflecting different patterns of activity observed in anterior and posterior hippocampal subregions as a function of event recency (other two-way interaction *p*s ≥ 0.138). However, and critically, this interaction must be qualified by a three-way interaction among the factors of pipeline, subregion, and temporal distance, *F*_(2,78)_ = 4.48, *p* = 0.041 (other three-way interaction *p*s ≥ 0.305). Unpacking this result revealed that significantly greater activity for recent (Today) than remote (5–10 year ago) activity was present in posterior, and not anterior, hippocampal regions, but only for the ME-ICA processed data [anterior hippocampus: *t*_(39)_ = 0.717, *p* = 0.478; posterior hippocampus: *t*_(39)_ = 2.91, *p* = 0.006] and not the SE data [anterior hippocampus: *t*_(39)_ = 0.876, *p* = 0.386; posterior hippocampus: *t*_(39)_ = 1.43, *p* = 0.160]. That is, a finding of temporally graded activity relied upon the improved denoising afforded by ME-ICA. No four-way interaction was observed, *F*_(1,39)_ = 0.05, *p* = 0.824.

Hippocampal response magnitudes for each processing stream and each temporal distance were then compared to the Picture Description baseline task. A time-limited role, as suggested by the “Standard Model of Consolidation” (Alvarez and Squire, 1994) would predict significantly greater activity for recent, but not remote, time periods when compared to Picture Description, whereas hypotheses that predict a continuous hippocampal involvement (e.g., Sekeres et al., 2018) would predict consistent activation over the baseline task for all retrieval conditions. Only four of the 16 contrasts were significant following Bonferroni correction (achieving *p* < 0.0031): two for ME-ICA datapoints associated with the Today condition [Right posterior hippocampus, *t*_(39)_ = 3.90, *p* = 0.0004; left posterior hippocampus, *t*_(39)_ = 3.38, *p* = 0.0016] and two SE datapoints, one for the 5–10 year ago condition [Right anterior hippocampus, *t*_(39)_ = 3.61, *p* = 0.0009] and one for the Today condition [left posterior hippocampus, *t*_(39)_ = 3.34, *p* = 0.0019] (Figures 1B,C). Applying an FDR correction (Benjamini and Yekutieli, 2001) instead of Bonferroni did not alter this pattern of results.

**FIGURE 3:**
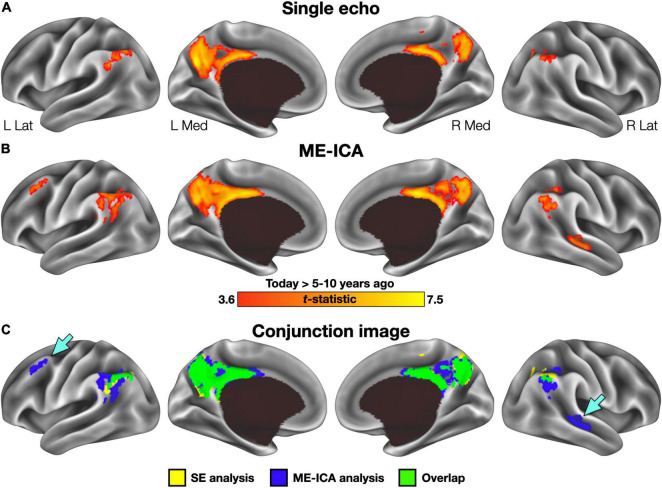
Voxelwise results for each preprocessing stream. **(A)** Regions exhibiting greater activity in the Today than 5–10 year ago condition following single-echo (SE) preprocessing included a large cluster in the posterior midline and inferior parietal lobule bilaterally. No clusters were identified with significantly greater activity for the 5–10 year ago condition. **(B)** Regions identified in the same analysis using multi-echo ICA (ME-ICA) data were larger and included several additional clusters in the right temporal and left frontal cortex. **(C)** A binarized conjunction image allows for easy visualization of the improved sensitivity offered by ME-ICA processing. Inset arrows identify significant clusters absent from the SE analysis.

Basic conclusions regarding hippocampal participation in recent and remote recall therefore differ between SE and ME-ICA processing streams—whereas, the ME-ICA data reflected a temporally graded and time-limited hippocampal role during recall, no clear support for any model was revealed by the SE analysis. However, beneficial effects of ME-ICA processing would be expected at the whole-brain level as well. Thus, a voxelwise contrast of activity related to the Today and 5–10 year ago conditions (paired-samples, two-tailed) was performed separately on the SE and ME-ICA data. The SE analysis identified a large cluster in medial parietal cortex, with local maxima in the mid/posterior cingulate cortex and bilaterally in the precuneus, as well as bilateral inferior parietal lobule clusters (Figure 3A and Table 2). Largely convergent results were obtained following ME-ICA processing, although commonly identified clusters were larger and additional significant clusters were identified in left frontal cortex and right lateral temporal cortex (Figures 3B,C and Table 2). No identified clusters were unique to the SE data.

**TABLE 2:** Regions identified in the voxelwise analysis of temporal distance effects for SE and multi-echo-ICA processing streams.

Region	*X*	*Y*	Z	Cluster size	Peak *t*-statistic
**SE processing stream**					
Medial parietal cortex	–4	–56	33	848	10.36
*Left precuneus*	–11	–66	30		10.36
*Mid-cingulate cortex*	2	–31	27		6.97
*Right precuneus*	12	–69	33		6.62
Left posterior inferior parietal lobule	–46	–62	40	50	4.77
Right posterior inferior parietal lobule	50	–55	43	20	4.94
Right intraparietal sulcus	34	–59	36	19	4.90
**ME-ICA processing stream**					
Medial parietal cortex	–1	–50	32	1039	7.85
*Mid-cingulate cortex*	–1	–31	27		7.42
*Right precuneus*	8	–69	37		7.85
*Left precuneus*	–11	–66	30		6.86
Left posterior inferior parietal lobule	–46	–56	39	148	5.18
Right posterior inferior parietal lobule	50	–56	36	75	5.21
Right superior temporal sulcus	56	–30	-4	60	5.06
Left middle frontal gyrus	–46	17	41	30	4.53

*Medial parietal subregions reflect discrete local maxima within a larger cluster. Coordinates refer to centers of mass in MNI152 space.*

## Discussion

In this report, we sought to investigate the practical benefits of an advanced fMRI denoising technique under naturalistic recall conditions. Given the rise in the number of experiments seeking to implement more naturalistic paradigms, the comparison of SE and ME-ICA approaches to data processing might help investigators optimize their sequence and processing pipeline selections. At least in the context of the current dataset, the addition of ME-ICA processing contributed positively to the interpretability of the obtained results.

The general efficacy of ME-ICA in denoising fMRI data has been documented elsewhere (Gonzalez-Castillo et al., 2016), particularly in investigations of functional connectivity (Kundu et al., 2012, 2013, 2017; Dipasquale et al., 2017; Power et al., 2018; Lynch et al., 2021). The purpose of this investigation was not to retread this same ground, but instead to focus on the practical utility of ME-ICA denoising in a naturalistic recall paradigm—an approach in which sophisticated denoising may be particularly important. In this case, ME-ICA-derived improvements appear to have been necessary for the findings obtained previously. This is most clearly demonstrated in the hippocampal results (Figure 2). Data processed in the SE pipeline failed to reveal any systematic differences as a function of temporal distance or in comparison to a non-autobiographical control task, and thus failed to support any specific model in the literature. In contrast, the ME-ICA data supported predictions of the Standard Model of Consolidation regarding both a temporally graded and time-limited role (Alvarez and Squire, 1994). Moreover, the benefits of ME-ICA were not restricted to the hippocampus. Voxelwise, whole-brain results also demonstrated the improved sensitivity of ME-ICA, both through identification of larger clusters than in the SE data (Figure 3C and Table 2), as well as the addition of several clusters not observed in the SE data. The additional clusters seem unlikely to be spurious findings, but rather captured processes relevant to the experiment: the cluster identified in left frontal cortex has previously been associated with autobiographical recall (with a neurosynth posterior probability of 0.79), whereas the lateral temporal cluster is typically associated with spoken language (posterior probability = 0.82).

Naturalistic paradigms, such as the overt cued recall approach described herein, provide promising avenues for researchers to ask questions that are difficult, if not impossible, to address using more limited and controlled laboratory tasks. However, with this flexibility comes additional concerns of how to make the best use of acquired data, including concerns regarding how one can be satisfied that data have been properly denoised. ME-ICA’s identification and regression of nuisance components provides a reliable means of improving timeseries quality (Figure 1). An important corollary of this improvement is that effects of interest should be detectable in fewer participants than in a traditional SE approach (or, as was the case here, improved sensitivity given the same number of participants). This improvement, it should be noted, comes despite a numeric loss of effective degrees of freedom during ICA denoising [for related discussion, see Gonzalez-Castillo et al. (2016), and Dipasquale et al. (2017)]. Denoising approaches such as ME-ICA may play increasingly important roles in data processing strategies and offer the potential to inform long-standing theoretical debates.

## Data Availability Statement

Publicly available datasets were analyzed in this study. These data can be found here: https://openneuro.org/datasets/ds003511/versions/1.1.1.

## Ethics Statement

The studies involving human participants were reviewed and approved by Institutional Review Board of the National Institute of Mental Health. The patients/participants provided their written informed consent to participate in this study.

## Author Contributions

AG conceptualized the study, processed the data, conducted the analyses, interpreted the results, and wrote the manuscript. AA and EG-A contributed to data processing and analysis. SG contributed to data processing, the interpretation of results, and made significant contributions to the manuscript. AM conceptualized the study, interpreted the results, and made significant contributions to the manuscript. All authors contributed to manuscript revision, read, and approved the submitted version.

## Conflict of Interest

The authors declare that the research was conducted in the absence of any commercial or financial relationships that could be construed as a potential conflict of interest.

## Publisher’s Note

All claims expressed in this article are solely those of the authors and do not necessarily represent those of their affiliated organizations, or those of the publisher, the editors and the reviewers. Any product that may be evaluated in this article, or claim that may be made by its manufacturer, is not guaranteed or endorsed by the publisher.

## Footnotes

1
https://www.mathworks.com/matlabcentral/fileexchange/27418-fdr_bh
